# The Geography of Diabetes in London, Canada: The Need for Local Level Policy for Prevention and Management

**DOI:** 10.3390/ijerph7052407

**Published:** 2010-05-19

**Authors:** Jordan W. Tompkins, Isaac N. Luginaah, Gillian L. Booth, Stewart B. Harris

**Affiliations:** 1 Centre for Studies in Family Medicine, Department of Family Medicine, Schulich School of Medicine & Dentistry, The University of Western Ontario, London, ON N6G 4X8, Canada; E-Mail: sharris1@uwo.ca; 2 Department of Geography, The University of Western Ontario, London, ON N6A 5C2, Canada; E-Mail: iluginaa@uwo.ca; 3 Institute of Clinical Evaluative Sciences, Toronto, ON M4N 3M5, Canada; E-Mail: boothg@smh.toronto.on.ca; 4 Department of Medicine and Health Policy, Management and Evaluation, University of Toronto, Toronto, ON M5T 3M6, Canada; 5 Department of Medicine and the Keenan Research Centre in the Li Ka Shing Knowledge Institute, St. Michael’s Hospital, Toronto, ON M5B 1W8, Canada

**Keywords:** diabetes mellitus, London, Ontario, public health, geography, socioeconomic determinants of health, health behaviours, health interventions

## Abstract

Recent reports aimed at improving diabetes care in socially disadvantaged populations suggest that interventions must be tailored to meet the unique needs of the local community—specifically, the community’s *geography*. We have examined the spatial distribution of diabetes in the context of socioeconomic determinants of health in London (Ontario, Canada) to characterize neighbourhoods in an effort to target these neighbourhoods for local level community-based program planning and intervention. Multivariate spatial-statistical techniques and geographic information systems were used to examine diabetes rates and socioeconomic variables aggregated at the census tract level. Creation of a deprivation index facilitated investigation across multiple determinants of health. Findings from our research identified ‘at risk’ neighbourhoods in London with socioeconomic disadvantage and high diabetes. Future endeavours must continue to identify local level trends in order to support policy development, resource planning and care for improved health outcomes and improved equity in access to care across geographic regions.

## 1. Introduction

Diabetes prevalence rates in Ontario have increased 69% in the past decade, rising from 5.2% in 1995 to 8.8% in 2005, exceeding the World Health Organization global projected prevalence of 6.4% by the year 2030 [1]. Individuals of lower socioeconomic status have higher rates of diabetes and worse outcomes [2–4], and adoption of an innovative political agenda designed to target this high risk population is necessary, specifically an agenda recognizing the unique contribution of socioeconomic determinants of health. Recent literature has placed an increased emphasis on reducing inequities across the socioeconomic hierarchy, and although individual risk factors must not be ignored, awareness of the many factors clearly outside of an individual’s control must be taken into consideration when designing innovative health policy [2,4]. Recognition of health inequalities must be embedded in political endeavours to ensure that they become a natural constituent of effective diabetes strategic initiatives [5].

Recent reports aimed at improving diabetes care in socially disadvantaged populations suggest that, regardless of the city, interventions must be tailored to meet the unique needs of the local community—specifically, the community’s *geography*. The view is that interventions adapted to fit local circumstances have greater potential to yield important benefits [5]. Health care and inequalities must be contextualized to place and a thorough assessment is critical to understanding the characteristics and needs of specific populations [6]. Spatial approaches tend to enable and empower health professionals and decision-makers with a unique set of informative tools for public health policy development [7]. In view of this, the primary objective of this work was to examine the spatial patterns of diabetes in London and identify high-risk (socioeconomic) areas that may be in unique need for community-based program planning and interventions. We hypothesized that there would be a strong spatial concordance between diabetes and socioeconomic determinants of health.

## 2. Methodology

### 2.1. Study Population

The study population consisted of all patients (N = 21,850; 49.5% female) diagnosed with diabetes in the city of London, Ontario, Canada (population = 352,395; 51.8% female), between 20 and 100 years of age between April 1, 2006 and March 31, 2007, and recorded in the Ontario Diabetes Database (ODD). The ODD uses a validated algorithm with a sensitivity of 86% and specificity of 97% as the gold standard for the diagnosis of diabetes in Ontario [8]. Age- and sex-adjusted diabetes prevalence rates were calculated to the overall Canadian population in 1991 (ages 20 years or older) using direct standardization and stratified by gender. The city of London has very similar population characteristics to Ontario on a whole and was deemed a reliable template for investigation in an ‘average’ Ontario city [9].

### 2.2. Study Design and Variables

We adopted a population-based ecological design, involving principal component analysis (PCA), geographic information systems (GIS) and spatial-statistical techniques to examine diabetes rates and socioeconomic variables aggregated at the census tract level. Census tracts were selected as the geographic unit of analysis since they are the smallest areas for which diabetes prevalence rates were available. Most research at the community and neighbourhood level use census or other political boundaries allowing straight-forward linkages with routinely collected area level data [9,10].

Variables were selected from the 2006 Census of Canada based on their established relationships in the literature as socioeconomic determinants of health [2,11–13]. Thirteen measures of socioeconomic determinants of health were obtained from the Statistics Canada, 2006 Census of Population (Table 1) [9].

A deprivation index was computed with a combination of three indicators (income, education, single-parenthood) chosen for their: (i) association with one of Townsend’s two forms of deprivation (social or material) [14]; (ii) acknowledged association to health [15]; and (iii) availability in the 2006 Canadian census data [9]. This methodology is supported and described in detail by Gilliland and Ross [15] and enables the creation of an index with a large amount of variation between neighbourhoods such that neighbourhoods with a deprivation index at each end of the spectrum will be amongst the 16% most or least deprived in London.

### 2.3. Analyses

Statistical analyses were performed using SPSS 15.0 and GeoDA™ 0.9.3a, and visualized using ArcGIS 9.2. We created thematic maps to visualize general trends of diabetes and socioeconomic determinants of health by census tracts in London. Patterns were visualized using chloropleth maps using the Jenks natural breaks algorithm Following the derivation of thematic maps, a correlation matrix of socioeconomic variables and diabetes prevalence rates was constructed. We then used PCA to reduce the dimensionality of the data set, while retaining any underlying variations that may have been present [16]. PCA transforms a large number of potentially correlated variables into a smaller number of extracted, uncorrelated variables called components. The principal component (PC) with the largest eigenvalue is the first principal component (PC1); the second largest eigenvalue represents the second principal component, and so on until the variation in the dataset is contained. Eigenvalues are additive, such that the linear combination of the variables accounts for the maximum total variability in the dataset. Successive principal components account for a proportion of the variability not accounted for by the preceding components. To further maximize variability, varimax rotation was employed [17]. For the un-stratified, general (both males and female) population, 13 socioeconomic determinants of health were computed; however when stratified by gender, 11 determinants were computed to determine the principal components. Rented dwellings could not be subdivided by gender, while visible minority status by gender was not available. Based on the fact that PC1 for males, females and both genders captured either the highest proportion, or a representative proportion of the variance for socioeconomic status, it was used in subsequent analysis. PC1 was divided into 3 equal intervals to represent census tracts characterized by Low, Middle and High socioeconomic status. Diabetes prevalence rates and principal components were overlaid to examine patterns of correspondence between high diabetes rates and socioeconomic determinants of health [7]. Relationships between determinants of health, PC1 values, the computed deprivation index and diabetes prevalence rates were evaluated using bivarate local indicator of spatial association (LISA). Bivariate LISA analyses calculate Local Moran’s (Ii) statistic to detect statistical spatial autocorrelation using an algorithm identifying spatial clusters of significantly similar or dissimilar values. Local Moran’s (Ii) statistic is defined by Anselin [18] as:

$$I_{i}=\frac{\left(x_{i}-x^{*})\Sigma_{j}w_{ij}(x_{j}-x^{*}\right)}{\Sigma_{i}{\left(x_{i}-x^{*}\right)}^{2}/n}$$

where *x_i_* is the observed value of *x* at the location *i*, x^*^ is the mean of *x*, *n* is the number of observations and *w_ij_* represents the nearest neighbour spatial connectivity matrix (in this case the first order nearest neighbour) which represents the strength of the linkage between *i* and *j* [18,19]. Positive values indicate spatial clustering of similar values (either high or low), and negative values a clustering of dissimilar values (for example, a location with high values surrounded by neighbours with low values). A 95% confidence interval was used to determine the statistical significance of spatial clustering. The outcomes of bivariate LISA are polygons based on five categories: *High-High, Low-Low, High-Low, Low-High,* and *Not Significant.* LISA statistics have three advantages: (i) identifying the existence of pockets or clusters; (ii) assessing assumptions of stationarity (*i.e.*, that spatial relationships are the same at all places in the study area); and (iii) determining distances beyond which no discernible spatial association exists [18]. Extending the aforementioned PCA with LISA allowed a more detailed description of the interrelationships between diabetes prevalence and socioeconomic deprivation, and overall, unearthing the complexity of neighbourhood health in the context of socioeconomic disadvantage and diabetes.

### 2.4. Ethical Approval

Ethical approval was obtained through the Research Ethics Board at Sunnybrook Health Sciences Centre.

## 3. Results

**Figure 1.**
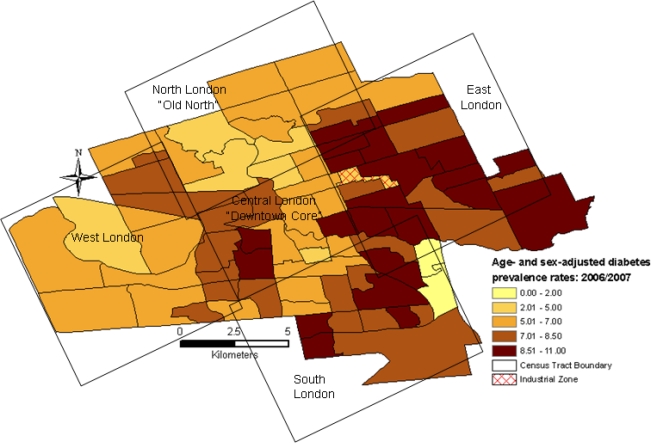
Age- and sex-adjusted diabetes prevalence rates per 100 persons aged 20+ in London, Ontario [2006/2007].

In 2006/2007, the overall age- and sex-adjusted diabetes prevalence rate in the city of London was 7.5 per 100 persons, with a maximum of 11.0 per 100 persons in select census tracts (compared to the Ontario rate of 8.8% in 2005) [20]. Diabetes prevalence rates were highest in areas known by residents as East London (Figure 1). Rates were also slightly high in the census tracts residing on the westerly side of the downtown core and select areas in South London. In contrast, diabetes prevalence rates were lowest in North and West London.

Following the derivation of thematic maps, a correlation matrix was constructed to explore correlations among the socioeconomic variables and diabetes prevalence rates. Correlations were examined at a significance level of *P*-value <0.05 and <0.01. A high degree of correlation existed between many of the variables (Table 1), and further analyses using PCA were conducted to reduce the dimensionality in the dataset while retaining any existing underlying variations.

Table 2 displays the PCA results for the un-stratified, general population. The PC matrix showed that the overall magnitude of the variable loadings was high and revealed a three dimensional matrix accounting for 75.8% of the total variance. The component loadings ranged in value from −1.0 to +1.0, and measured the relationship of the original variables with each factor (numbers in bold font denote components that load highly on each principal component). Principal component 1 (PC1) explained the highest percentage of the variance with 35.0%, and was characterized by a high percentage of the population falling below Statistics Canada’s low income cut-offs (LICO) for both families and individuals, high proportion of rental properties, high unemployment, moderately high percentage of recent immigrants and individuals not in the labour force, and low average and median household income.

Table 2 displays the PCA results for the un-stratified, general population. The PC matrix showed that the overall magnitude of the variable loadings was high and revealed a three dimensional matrix accounting for 75.8% of the total variance. The component loadings ranged in value from −1.0 to +1.0, and measured the relationship of the original variables with each factor (numbers in bold font denote components that load highly on each principal component). Principal component 1 (PC1) explained the highest percentage of the variance with 35.0%, and was characterized by a high percentage of the population falling below Statistics Canada’s low income cut-offs (LICO) for both families and individuals, high proportion of rental properties, high unemployment, moderately high percentage of recent immigrants and individuals not in the labour force, and low average and median household income. PC1 was referred to as the *low income, high rental, unemployed* component. The second component (PC2) explained 24.3% of the total variance and showed high percentages of lone parents, low education (high proportion of individuals lacking a high school education, with correspondingly low proportion with a university education), moderately high percentage of individuals who do not speak French or English, and moderately low average and median household income. PC2 was referred to as the *low income, low education, lone parent* component. Principal component 3 (PC3) explained 16.5% of the total variance. PC3 portrayed a high percentage of the population considered a visible minority, high percentage of individuals who do not speak French or English, moderately high percentage who are a recent immigrant, moderately high percentage falling below Statistics Canada’s LICO (families only), and moderately high percentage of lone parents. PC3 was termed the *low income (families), visible minority, recent immigrant with no knowledge of French or English* component.

The above described three components represent the underlying socioeconomic variables that assist in describing the un-stratified, general population in the city of London. The communalities are high (with the exception of the variable ‘not in labour force’), indicating that the three-component structure is an appropriate way of reducing the original socioeconomic determinants of health. Principal component analysis for the stratified (by gender) populations revealed slightly different PC values and are presented in Table 3.

Principal components 1, 2 and 3 were similar in both males and females, suggesting that similar underlying processes affect males and females in the London area (noted exceptions are detailed below). The PC matrix for males revealed a three dimensional matrix accounting for 62.6% of the total variance. Principal components 1, 2 and 3 accounted for 21.7% and 21.1% and 19.8% of the total variance respectively, suggesting that the three principal components account for a similar proportion of the variance in socioeconomic status for males in London.

The PC matrix for females similarly revealed a three dimensional matrix accounting for 68.8% of the total variance; however only principal components 1 and 2 accounted for the higher proportion of the variance (28.7% and 26.4% of the total variance respectively). Principal component 3 accounted for 13.7% of the total variance. For both males and females, PC1 portrayed very low education, moderately high single parenthood, moderately high number of families falling below Statistics Canada’s LICO, and moderately high percentage of non-English or French speaking residents. For females, PC1 was also characterized by moderately low income and moderately high proportion not in the labour force. As such, PC1 for the stratified population was referred to as the *low education, single parent with moderately low income* component. PC2 had a moderately high percentage of the population who were unemployed, high (individuals) and moderately high (families) falling below Statistics Canada’s LICO, high recent immigrant status with moderately high percentage of individual not in the labour force. PC2 also included a moderately high percentage of females with low average and median household income. PC2 was termed the *low income, unemployed, recent immigrant* component. PC3 for males was characterized by high income and moderately high single parenthood, while PC3 for females was characterized by moderately high income and moderately high proportion not in the labour force. PC3 was referred to as the *high income, single parent* component for males and *high income, non-labour force* component for females.

Following the principal component analysis, LISA statistics were computed to further explore clusters revealed in the PCA and to deepen our understanding of the underlying interrelationships between socioeconomic determinants of health and diabetes prevalence rates in London. LISA was performed separately using PC1, the deprivation index and individual socioeconomic determinants of health as input variables. Results of the LISA analysis have been selected to demonstrate the variation in key findings.

**Figure 2.**
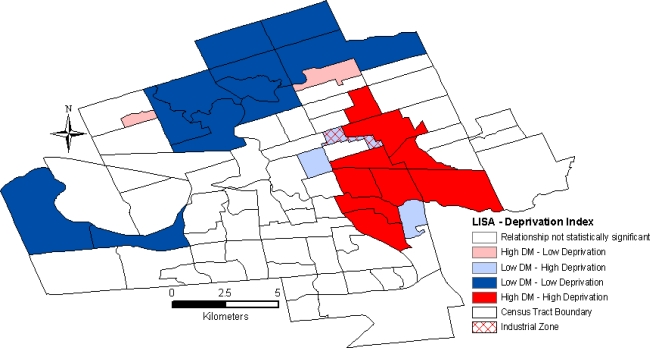
Spatial relationship between diabetes prevalence rates [2006/2007] and deprivation index [2006] in London, Ontario for the general, un-stratified population.

LISA analyses of PC1 values for the stratified population and the deprivation index for the general, un-stratified population, revealed a similarly high spatial concordance between diabetes prevalence rates and socioeconomic determinants of health (Figure 2 shows the results of the LISA analysis of the age- and sex-adjusted diabetes prevalence rates and deprivation index). The spatial relationships illustrate a distinct pattern of high diabetes rates associated with a higher deprivation in East London and low diabetes rates and low deprivation in North and West London.

**Figure 3.**
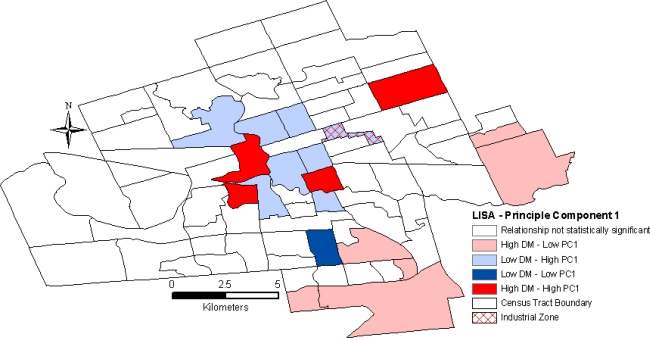
Spatial relationship between diabetes prevalence rates [2006/2007] and principle component 1 [2006] in London, Ontario for the general, un-stratified population.

In contrast, LISA analysis of PC1 values for the general population revealed an overlap of high PC1 values (low socioeconomic status) and low age- and sex-adjusted diabetes prevalence rates in the downtown core (Figure 3).

## 4. Discussion

The association between the health status of individuals and their position on the socioeconomic hierarchy is evident in the literature and has been widely demonstrated in numerous populations [4,10,21,22]. Everson and colleagues refer to this as a social gradient or dose-response relationship between socioeconomic status and health, and individuals on the low end of the spectrum consistently suffer a disproportionate share of negative health consequences than the rest of a population [22].

Results suggest that as local health and policy planners strive to develop strategies poised at diabetes prevention and management, the city of London can be characterized into five distinct neighbourhoods. Findings indicate that East London, on the whole, can be described as socially and ethnically diverse, with high diabetes prevalence rates and high socioeconomic deprivation (although the male population displayed slightly less social disadvantage and diversity than females, a finding consistent with the literature on gender differences in health) [4]. North and West London, regions habitually labelled as affluent by residents of the city, exhibited moderate to low social diversity, low diabetes prevalence rates and high socioeconomic status. One exception to this pattern was a select few census tracts in the north-west corner of the city with moderately high diabetes prevalence and low socioeconomic status. Specifically, this region can be characterized by a high density of elderly individuals (≥65 years of age) which may account for the uncharacteristically high diabetes prevalence rates. Analysis of Central London revealed low social diversity, low diabetes prevalence rates, and high socioeconomic deprivation, while select census tracts immediately surrounding the downtown Core exhibited paralleled high social deprivation with conversely high diabetes prevalence rates. Lastly, South London can be characterized by high social and ethnic diversity, high diabetes prevalence rates and low socioeconomic status. Similar to East London, South London displayed higher rates of socioeconomic depravity for the female population.

Previous research in Hamilton, Ontario [7] indicated that PCA, LISA and GIS can be used as complementary tools for improving our understanding of socioeconomic determinants of health at the local level. The results presented here suggest a strong spatial concordance between socioeconomic determinants of health and diabetes rates in London. One exception to this pattern was the existence of low diabetes rates and low socioeconomic status in Central London, a finding inconsistent with the majority of the literature [4,10,21,22] and potentially explained by the incorporation of ‘rental properties’ for the general population in PC1. The pattern of rental properties corresponded to areas surrounding The University of Western Ontario and the downtown core, and may be an imprecise marker for determinants of health for the population on a whole in London with a high turn-over rate of residents in the area (specifically if the transitional population of students participate in the census). Patterns in Central London highlight the importance of understanding the characteristics of a city when interpreting results, and future research could help to elucidate these findings.

Similar to other contexts, diabetes disproportionately affects individuals of lower socioeconomic status, specifically those in lower income, lower education, high visible minority and high recent immigrant brackets [2,23,24]. It also appears to be particularly detrimental for women, a finding consistent with the literature [4]. This research provides local policy makers with a tool to guide public health policy initiatives and resource planning for the prevention and effective management of diabetes.

### 4.1. Policy Options

Moving forward in public health policy and planning, this research recommends an intentional two-tiered approach to combating diabetes as a chronic disease including: (i) tailored local level interventions for individuals, and (ii) community based policy initiatives.

For a diabetes intervention to be successful, it must be tailored to meet the needs of the community [6]. Contextualizing this in London, diabetes resources could, ideally, be customized for each of the identified and unique areas (neighbourhoods). More deprived areas could be provided with increased resources to manage the population at risk of developing the disease or its complications [6,25]. The Diabetes Education Centre at St. Joseph’s Health Care in London hosts a variety of educational initiates aimed at bringing diabetes education into the community. These initiatives, termed *Diabetes London*, are held at the Central Library situated in the downtown Core. *Diabetes London* boasts the success of moving diabetes education out of a hospital setting into the community, and similar strategies could be used to target East and South London, encouraging residents by making diabetes interventions readily accessible, available, and culturally and linguistically tailored to target the non-English and French speaking, new immigrant, visible minority population. This initiative fits into the Southwestern Ontario’s Local Health Integration Network (LHIN) strategy of redirecting existing diabetes care and education into areas identified with high need [26].

At a community level, researchers have begun to highlight the physical environment in city and area planning as one approach to combat rising obesity and diabetes rates. Research on obesogenic environments (environments that encourage physical inactivity and poor eating habits), high-risk generations of children, and parent’s preferences for parks in the city of London stress the importance of neighbourhoods for one’s physical, social and mental well-being, and incorporating both the *natural* and *built* environment in city and area planning [27–30].

London has also been home to recent research on food deserts, and findings indicate that low socioeconomic residents of Central London have the poorest access to supermarkets in the city. In addition, urban food deserts were found in Central and East London, with spatial inequalities in access to supermarkets increasing over time since 1961 [31]. The presence of food deserts in areas of low socioeconomic status present the challenge of simply getting to a grocery store to access affordable, nutrient rich food, requiring the availability of a vehicle or bus and the additional travelling time [31]. Community infrastructure planning to position new stores in locales identified as ‘food deserts’ could aid in reducing inequities in access in certain regions of the city, and future research could examine the relationships between the built environment and diabetes in the city of London.

Researchers stress the importance that “economic policy is public health policy” (p. 809) when health behaviours are intertwined with social hierarchy [32]. An economic approach recognizes the problem at the societal and public health level, and supports the pivotal role of the government in initiatives such as subsidies to relieve the burden of purchasing healthy foods [33,34]. The 2008 report by the World Health Organization Commission on Social Determinants of Health highlighted governmental action as the centerpiece for closing the gap between the rich and the poor, stating that organizations dedicated to reducing health disparities do not have the capacity to compensate for the lack, or withdrawal, of federal and/or provincial assistance. This concept was reiterated by the 2008 report of the Canadian Senate Subcommittee on Population Health [35]. Eloquently stated by Geoffrey Rose in 1992, “the primary determinants of disease are mainly economic and social, and therefore its remedies must also be economic and social. Medicine and politics cannot and should not be kept apart” [36].

### 4.2. Methodological Limitations

It is important to address a number of methodological limitations in this research. Firstly, the use of census tracts to measure area level influences on health are supported and appropriate for investigation; however these may be unsuitable for drawing conclusive judgements if the census tracts do not align with the geographical distribution of factors linking place and health [13,24]. Furthermore, although boundary lines were defined for the purposes of simplifying explanations, for example ‘North London’, these are not designed to reflect exact borders between sub-regions of the city. Regardless of the area level measures used as proxies for individuals, care must be taken to avoid ecological fallacy, since group level data is being used to make inferences at the individual level. The use of measures based on geographic areas rather than individual conditions causes the implicit assumption of equality between people living in the same area, and care must be taken in the interpretation of results. Heterogeneity within census tracts was not examined in this research; however significant literature suggests that both the average and spread of a variable of interest should be examined to more fully understand neighbourhood social and contextual factors affecting inequality [10].

Secondly, interpretation of PCA is subjective, and although all three principal components reflect the variability between neighbourhoods and contribute individually in the area level analysis, only PC1 was used in the LISA analysis. Specifically when the data was stratified by males and females, PC1 and PC2 contributed an equal percentage of variance in the dataset (including PC3 for males), suggesting that no one principal component explained the majority of the variance in the population [7].

### 4.3. Future Research

The Ontario Ministry of Health and Long Term Care (MOHLTC) recently launched a new comprehensive Diabetes Strategy to inform diabetes care and prioritize diabetes treatment and innovative techniques in primary care [26]. The Diabetes Strategy includes the inauguration of a Clinical Diabetes Registry in 2010 in Southwestern Ontario’s Local Health Integration Network (LHIN) followed by provincial integration by 2012. One of the biggest challenges in health geographical research is the lack of individual-level data [37], necessitating a reliance by policy makers and planners on ecological study designs to assess the geography of health and illness. Linking the clinical data from the Diabetes Registry with determinants of health may provide valuable insight and findings from future research will be well positioned for impacting diabetes policy in London.

## 5. Conclusions

Diabetes disproportionately affects individuals at the lower end of the socioeconomic hierarchy, and considerable literature has shown that effective diabetes health policy and interventions are contingent on the ability of policy makers to tailor the intervention to meet the unique needs of high-risk individuals and communities. Using innovative analytic approaches including geographic information systems and through the creation of a deprivation index, our research demonstrates that health disparities exist across the city of London, and pinpoints specific areas that would benefit from tailored diabetes health services and preventative interventions. Due to the human and economic burden of the disease, this research is important for focusing efforts on appropriate preventative care and reducing risk factors for complications. Although individual behavioural risk factors must not be ignored, we must be aware of the many factors clearly outside of an individual’s control that must be taken into consideration in planning for effective health policy. The concept of victim-blaming will not allow our health care system to progress towards combating the epidemic, and reducing inequities across the socioeconomic spectrum. Future endeavours must continue to identify local level trends and patterns in order to provide evidence to support policy development, resource planning and care for improved health outcomes and improved equity in access to care.

## Figures and Tables

**Table 1.** Correlation matrix for general (un-stratified) population.

	X_1_	X_2_	X_3_	X_4_	X_5_	X_6_	X_7_	X_8_	X_9_	X_10_	X_11_	X_12_	X_13_	X_14_
Single Parent, X_1_	1.000	0.099	0.227*	0.471**	0.194*	0.397**	0.136	0.503**	–0.560**	0.568**	0.262**	–0.454**	–0.518**	0.683**
Recent Immigrant, X_2_		1.000	0.627**	0.226*	0.566**	0.320**	0.227*	–0.160	0.265**	0.481**	0.499**	–0.344**	–0.291**	–0.049
Visible Minority, X_3_			1.000	0.550**	0.241*	0.279**	0.150	–0.122	0.178	0.449**	0.319**	–0.128	–0.130	0.026
No French/English, X_4_				1.000	0.119	0.250*	0.197*	0.347**	–0.288**	0.420**	0.206*	–0.228*	–0.257**	0.367**
Rented Dwellings, X_5_					1.000	0.514**	0.455**	0.264**	–0.053	0.647**	0.797**	–0.829**	–0.654**	0.252*
Unemployment, X_6_						1.000	0.305**	0.154	–0.128	0.583**	0.694**	–0.525**	–0.458**	0.327**
Not in Labour Force, X_7_							1.000	0.255**	–0.006	0.284**	0.330**	–0.376**	–0.230*	0.193
Lacking High School, X_8_								1.000	–0.820**	0.359**	0.180	–0.545**	–0.649**	0.805**
University Educated, X_9_									1.000	–0.256**	–0.014	0.390**	0.572**	–0.860**
LICO^a^ – families, X_10_										1.000	0.806**	–0.677**	–0.621**	0.418**
LICO^a^ – private, X_11_											1.000	–0.778**	–0.609**	0.213*
Median Income, X_12_												1.000	0.852**	–0.537**
Average Income, X_13_													1.000	–0.668**
DM Prevalence, X_14_														1.000

* Correlation is statistically significant at the *P*-value < 0.05.

** Correlation is statistically significant at the *P*-value < 0.01.

a LICO – living below Statistics Canada low income cut-off.

**Table 2.** Principal component analysis for general (un-stratified) population (N = 352,395).

	**Component**			

**Variables**	**1**	**2**	**3**	**Communalities^a^**
Single parent	0.217	**0.723**	**0.383**	0.716
Recent Immigrant	**0.554**	0.326	**0.553**	0.720
Visible Minority	0.195	−0.157	**0.902**	0.876
Language	0.027	**0.424**	**0.766**	0.767
Rented Dwelling	**0.936**	0.031	0.072	0.881
Unemployment	**0.703**	0.147	0.213	0.561
Not in Labour Force	**0.502**	0.056	0.040	0.257
Education (lacking high school)	0.215	**0.905**	−0.056	0.868
Education (university or more)	−0.018	**−0.934**	0.078	0.879
Low income–LICO(families)	**0.703**	0.302	**0.446**	0.783
Low income–LICO(individuals)	**0.900**	0.025	0.187	0.846
Median household income	**−0.849**	**−0.420**	−0.013	0.898
Mean household income	**−0.681**	**−0.589**	−0.051	0.813

Eigenvalue^b^	4.550	2.510	1.520	
Percentage variance explained	35.0	24.3	16.5	75.8

a Communality is the proportion of a variable’s variance explained by the retained factors.

b Eigenvalue does not apply to communalities.

**Table 3.** Principal component analysis stratified by males (N = 169,854) and females (N = 182,541).

	**Component**							

**Variables**	**1**		**2**		**3**		**Communalities^a^**	

	**Male**	**Female**	**Male**	**Female**	**Male**	**Female**	**Male**	**Female**
Single parent	**0.475**	**0.660**	0.254	0.295	**0.460**	–0.160	0.502	0.548
Recent Immigrant	–0.297	–0.209	**0.757**	**0.785**	0.161	0.006	0.687	0.660
Language	**0.372**	**0.421**	0.248	0.306	–0.199	0.036	0.240	0.272
Unemployment	0.320	0.154	**0.606**	**0.658**	–0.244	0.008	0.529	0.457
Not in Labour Force	–0.051	**0.375**	**0.570**	**0.395**	–0.094	**0.684**	0.337	0.765
Education (lacking high school)	**0.910**	**0.929**	0.039	0.076	–0.004	0.031	0.830	0.869
Education (university or more)	**–0.921**	**–0.913**	0.093	0.119	–0.083	0.195	0.863	0.886
Low income–LICO (families)	**0.351**	**0.387**	**0.554**	**0.690**	0.126	–0.228	0.446	0.677
Lowincome–LICO (individuals)	0.171	0.235	**0.776**	**0.837**	0.010	–0.126	0.632	0.772
Median household income	–0.030	**–0.444**	–0.062	**–0.413**	**0.948**	**0.674**	0.903	0.823
Mean household income	–0.003	**–0.488**	–0.049	**–0.395**	**0.954**	**0.669**	0.913	0.842

Eigenvalue^b^	2.387	3.160	2.214	2.900	1.877	1.500		
Percentage variance explained	21.7	28.7	21.1	26.4	19.8	13.7	62.6	68.8

a Communality is the proportion of a variable’s variance explained by the retained factors.

b Eigenvalue does not apply to communalities.

## References

[1] Booth GL Hux JE Fang J Chan BT Time trends and geographic disparities in acute complications of diabetes in Ontario, Canada Diabetes Care 2005 28 1045 1050 15855565 10.2337/diacare.28.5.1045

[2] Creatore MI Gozdyra P Booth GL Ross K Glazier RH Socioeconomic status and diabetes Neighbourhoods Environments and Resources for Healthy Living—A Focus on Diabetes in Toronto: An ICES Practice Atlas Glazier RH Booth GL Institute for Clinical Evaluative Sciences Toronto, ON, Canada 2007 36 56

[3] Robert SA Community-level socioeconomic status effects on adult health J. Health Soc. Behav 1998 39 18 37 9575702

[4] Yu VL Raphael D Identifying and addressing the social determinants of the incidence and successful management of type 2 diabetes mellitus in Canada Can. J. Public Health 2004 95 366 368 15490927 10.1007/BF03405148 PMC6975724

[5] Hartwell H Health inequalities—fair inequality? Perspect. Public Health 2009 129 194 19788152 10.1177/17579139091290050101

[6] Glazier RH Bajcar J Kennie NR Willson K A systematic review of interventions to improve diabetes care in socially disadvantaged populations Diabetes Care 2006 29 1675 1688 16801602 10.2337/dc05-1942

[7] Luginaah I Jerrett M Elliott S Eyles J Parizeau K Birch S Abernathy T Veenstra G Hutchinson B Giovis C Health profiles of Hamilton: spatial characterisation of neighbourhoods for health investigations GeoJournal 2001 53 135 147

[8] Hux JE Ivis F Flintoft V Bica A Diabetes in Ontario: Determination of prevalence and incidence using a validated administrative data algorithm Diabetes Care 2002 25 512 516 11874939 10.2337/diacare.25.3.512

[9] 2006 Community Profiles Catalogue No 92-591-XWE Statistics Canada Ottawa, ON, Canada 2007

[10] Pickett KE Pearl M Multilevel analyses of neighbourhood socioeconomic context and health outcomes: a critical review J. Epidemiol. Community Health 2001 55 111 122 11154250 10.1136/jech.55.2.111 PMC1731829

[11] Birch S Jerrett M Eyles J Heterogeneity in the determinants of health and illness: the example of socioeconomic status and smoking Soc. Sci. Med 2000 51 307 317 10832576

[12] Jerrett M Eyles J Cole D Socioeconomic and environmental covariates of premature mortality in Ontario Soc. Sci. Med 1998 47 33 49 9683377 10.1016/s0277-9536(98)00008-2

[13] Wilkinson RG Unhealthy Societies: the Afflictions of Inequality Routledge London, UK 1996

[14] Townsend P Phillimore P Beattie A Health and Deprivation: Inequality and the North Croom Helm Ltd Kent, UK 1988

[15] Gilliland J Ross N Opportunities for video lottery terminal gambling in Montreal: an environmental analysis Can. J. Public Health 2005 96 55 59 15682698 10.1007/BF03404019 PMC6976282

[16] Jolliffe IT Principal Component Analysis Springer-Verlag New York, NY, USA 1986

[17] Kaiser HF The varimax criterion for analytic rotation in factor analysis Psychometrika 1958 23 187 201

[18] Anselin L Local indicators of spatial association LISA Geogr. Anal 1995 27 94 115

[19] Fotheringham AS Brunsdon C Charlton N Quantitative geography Sage London, UK 2000

[20] Lipscombe LL The growing prevalence of diabetes in Ontario: are we prepared? Healthc. Q 2007 10 23 25 17626544 10.12927/hcq..18920

[21] Kawachi I Kennedy BP Health and social cohesion: why care about income inequality? BMJ 1997 314 1037 1040 9112854 10.1136/bmj.314.7086.1037 PMC2126438

[22] Everson SA Maty SC Lynch JW Kaplan GA Epidemiologic evidence for the relation between socioeconomic status and depression, obesity, and diabetes J. Psychosom. Res 2002 53 891 895 12377299 10.1016/s0022-3999(02)00303-3

[23] Hux JE Tang M Patterns of prevalence and incidence of diabetes Diabetes in Ontario: An ICES Practice Atlas Hux JE Booth GL Slaughter PM Laupacis A Institute for Clinical Evaluative Sciences Toronto, ON, Canada 2003 2 15

[24] Ross NA Tremblay SS Graham K Neighbourhood influences on health in Montreal, Canada Soc. Sci. Med 2004 59 1485 1494 15246176 10.1016/j.socscimed.2004.01.016

[25] Buckingham K Freeman PR Sociodemographic and morbidity indicators of need in relation to the use of community health services: observational study BMJ 1997 315 994 996 9365299 10.1136/bmj.315.7114.994 PMC2127650

[26] South West LHIN Transforming Health Care: A Community Approach; 2007–2008 Annual Report South West Local Health Integration Network London, ON, Canada 2008

[27] Harrington DW Elliott SJ Weighing the importance of neighbourhood: a multilevel exploration of the determinants of overweight and obesity Soc. Sci. Med 2009 68 593 600 19095339 10.1016/j.socscimed.2008.11.021

[28] He M Beynon C Prevalence of overweight and obesity in school-aged children Can. J. Diet. Pract. Res 2006 67 125 129 16968560 10.3148/67.3.2006.125

[29] He M Evans A Are parents aware that their children are overweight or obese? Do they care? Can. Fam. Physician 2007 53 1493 1499 17872878 PMC2234629

[30] Tucker P Gilliland J Irwin JD Splashpads, swings, and shade: parents’ preferences for neighbourhood parks Can. J. Public Health 2007 98 198 202 17626384 10.1007/BF03403712 PMC6976133

[31] Larsen K Gilliland J Mapping the evolution of ‘food deserts’ in a Canadian city: supermarket accessibility in London, Ontario, 1961–2005 Int. J. Health Geogr 2008 7 16 18423005 10.1186/1476-072X-7-16 PMC2387138

[32] Lynch JW Kaplan GA Salonen JT Why do poor people behave poorly? Variation in adult health behaviours and psychosocial characteristics by stages of the socioeconomic lifecourse Soc. Sci. Med 1997 44 809 819 9080564 10.1016/s0277-9536(96)00191-8

[33] Drewnowski A Specter SE Poverty and obesity: the role of energy density and energy costs Am. J. Clin. Nutr 2004 79 6 16 14684391 10.1093/ajcn/79.1.6

[34] Adler NE Newman K Socioeconomic disparities in health: pathways and policies Health Aff 2002 21 60 76 10.1377/hlthaff.21.2.60 11900187

[35] Kirkpatrick SI McIntyre L The chief public health officer’s report on health inequalities: what are the implications for public health practitioners and researchers? Can. J. Public Health 2009 100 93 95 19839281 10.1007/BF03405513 PMC6973718

[36] Rose G The Strategy of Preventive Medicine Oxford University Press Oxford, UK 1992

[37] Luginaah I Health geography in Canada: where are we headed? Can. Geogr 2009 53 89 97

